# Taking More Than a Fair Share? The Migration of Health Professionals from Poor to Rich Countries

**DOI:** 10.1371/journal.pmed.0020109

**Published:** 2005-05-31

**Authors:** Delanyo Dovlo

## Abstract

The migration of health professionals from developing countries undermines the ability of these countries to meet the Millennium Development Goals.

## Brain Drain: A Global Health Problem

The international migration of health workers, especially of physicians and nurses but also increasingly of other health workers, has become a major global health concern. Recent meetings, such as the World Health Assembly of 2004 [1] and the High-Level Forum on the Millennium Development Goals in December 2004, as well as a number of publications have highlighted the severe shortage of health personnel in poorer parts of the world and the rise in demand for health workers in rich countries. The 2005 World Health Assembly, being held this month, is expected to discuss how to limit the adverse effects of the migration and to promote fairer recruitment tactics by developed countries as a follow-up to a resolution passed in 2004.

There is now considerable interest in measuring and managing the migratory flow of health workers; in seeking reparations, payments or remittances; and in training “substitute health workers”—groups who have taken on jobs, functions, and roles that are normally the tasks of internationally recognized professionals such as doctors, nurses, and pharmacists [2]. Indeed, words such as “slavery” and “human rights” underlie the debate's emotional underpinnings (such words have appeared, for example, in listserv discussions about health worker migration).

**Figure 1:**
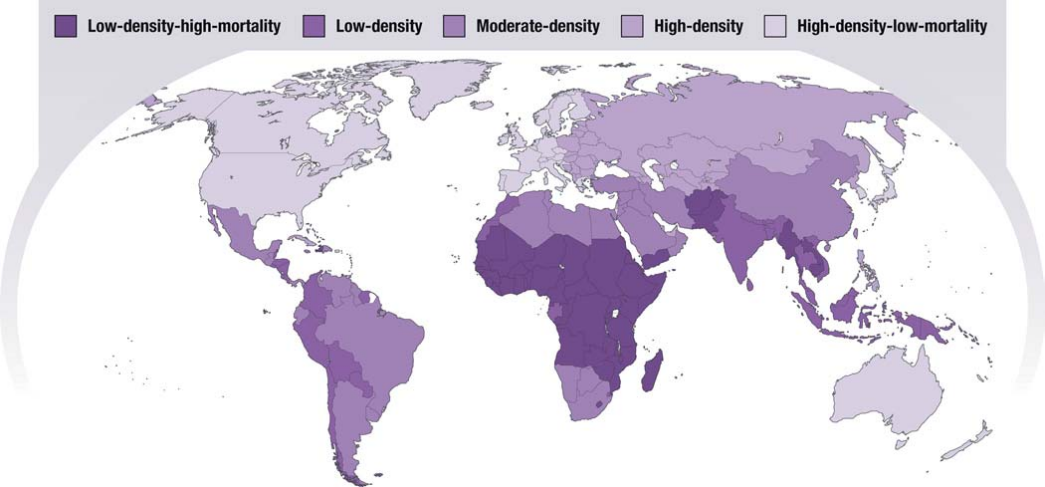
Global Variation in the Density of Health Workers In a report by the Joint Learning Initiative, 186 countries were designated as having low, medium, and high worker density clusters (below 2.5, between 2.5 and 5.0, and above 5.0 workers per 1,000 population, respectively), with the low- and high-density clusters further subdivided according to high and low under-five mortality [9]. Among low-density countries, 45 are in the low-density/high-mortality cluster; these are predominantly sub-Saharan countries experiencing rising death rates and weak health systems. (Illustration: Giovanni Maki, adapted from [9])

Though some migration occurs between rich countries (and also between poor countries), most of the migration of health professionals is occurring from countries with physician densities of about 17 per 100,000 population to countries with densities of 300 per 100,000 population (see p. 16 of [3]). This is a good example of the “inverse care law”—that countries that need the most health care resources are getting the least (Figure 1). Why does this migration occur when there appears to be a glut of physicians in the recipient countries? One of the reasons is that pay levels are up to 24 times higher in recipient countries than they are in source countries [4].

Countries such as India, the Philippines, and Nigeria—highly populated countries that train large numbers of health professionals and have a long-standing remittance culture, in which professionals working outside the country send money back home to relatives—have produced doctors and nurses for “export.” However, demand for different kinds of health professionals can fluctuate. For example, recent rises in demand for nurses in the United States led to reports of immigrant doctors in Florida who took up nursing to get into the job market [5]. This doubly wastes the resources poor countries invest into training physicians; indeed, other anecdotes suggest that many immigrant physicians and nurses take on jobs completely unrelated to their training.

## The Impact of Migration

For source countries with few physicians, the loss of even a single doctor often has a major impact on the health service [6]. In their analysis of the impact of the migration of health professionals, Martineau and colleagues state: “The ultimate losers tend to be health services (and their users) in the remoter rural areas, as they come lowest in the pecking order of people's preferred working location” [6]. And since it is the poorest citizens who live in the remoter areas, say the authors, it is they who are affected most by migration.

Often, health professionals leave to undertake training only obtainable (or seductively offered as “scholarships”) in rich recipient countries. Several years later, the metamorphosis from student to migrant is complete, but the migrant professionals may remain on payrolls in their home countries for several years. Thus, barely affordable initiatives towards capacity-building result instead in further losses of capacity.

Policy-makers in sub-Saharan Africa must feel helpless when they are completely unable to match either the remuneration or the working conditions found in recipient countries. A self-sustaining cycle ensues: as more and more physicians migrate from a country, they create an environment (a “home from home”) that entices newer migrants from the same country. The helplessness is reinforced by a lack of information on workforce losses for policy-making.

## A New Study on Physician Migration

A new study by Hagopian and colleagues analyzes the numbers, characteristics, and trends in the migration to the United States of physicians trained in sub-Saharan Africa [7]. The US is perhaps the world's largest “consumer” of health workers from the developing world.

The authors did a cross-sectional study using the 2002 American Medical Association Physician Masterfile, which contains detailed information on all 771,491 active physicians who were licensed to practice medicine in the year 2002 (excluding those physicians employed by federal entities such as the Veterans Administration, federal prisons, or the military). The authors reviewed these data for all physicians in the US who received their training in sub-Saharan Africa.

What they found was that more than 23% of America's 771,491 physicians received their medical training outside the country, mostly (64%) in low-income or lower-middle-income countries. A total of 5,334 physicians from sub-Saharan Africa are in that group, a number that represents more than 6% of the physicians practicing in sub-Saharan Africa now. Nearly 86% of these Africans practicing in the US originate from only three countries: Nigeria, South Africa, and Ghana. Of these, 79% were trained at just ten medical schools.

## Implications of the Study

This study suggests that selection of particular types of medical graduates is occurring, either for their training or language skills or, as mentioned earlier, by the presence of previous migrants from the same schools. Hagopian and colleagues state that there are several federal agencies and state health departments who “sponsor” physicians who have completed their residency training in the US on a “student” (J-1) visa. These sponsorships allow physicians who are foreign nationals to gain approval from the State Department and US Citizenship and Immigration Services to waive J-1 visa requirements that would otherwise require them to return to home countries for at least two years. In exchange for this waiver, physicians find employment with a health agency or private physician in a health professional shortage area. Such waivers raise the possibility of deliberate selection of the best graduates.

A common argument against any restriction of migration of health workers is that the right to live anywhere one chooses is a basic human right. This argument is particularly used by recipient countries. But even when we acknowledge such human rights concerns, the study by Hagopian and colleagues rightly notes that the migration tide subverts the development potential of poor countries. Migration further undermines the right of the people who contributed to the training of their country's health professionals to benefit directly from their investment.

The study also alludes to a lack of data about migration from poorer countries, such that accurate data on who has left, and to which countries they emigrated, are more easily obtained in the recipient country. Departures from the workforce need to be more properly documented in source countries. This documentation may be particularly difficult for countries that pay “ghost workers” (names on a payroll for employees that do not actually exist) [8], obscuring the real losses from the health system.

Physician shortages exist worldwide, but this study shows that the numbers of medical schools are very low in sub-Saharan Africa. Conventional medical training in tertiary hospitals is very expensive. Yet, instead of training new types of workers to match local needs, many countries in sub-Saharan Africa continue to adhere to such training out of professional pride—they are reluctant to been seen to be using less than the “best” type of health worker. Furthermore, professionals in these countries wish to retain reciprocal recognition of their qualifications by the developed world—another obstacle to training new types of workers, who may not be internationally recognized. Professional associations and regulatory councils have resisted or limited the introduction of substitute health workers. Production of enrolled and auxiliary nurses, for example, was banned in some of the poorest countries (such as Zambia and Ghana), ostensibly to enhance the status of the professions even as health and economic indices receded and remaining professionals fled.

## International Arrangements to Manage Flows?

Although there is a moral argument that richer recipient countries should help to mitigate migration, this argument is usually derided even by source countries, perhaps because they are powerless and are often dependent on the same recipient countries for aid.

Unlike the United Kingdom, the US has a multifaceted health system, which makes it difficult to imagine a situation in which it is feasible to agree on control of physician migration from countries that have little political or economic clout. Such source countries have little influence when it comes to negotiating over the poaching of their health professionals by powerful countries such as the US.

**Figure 2:**
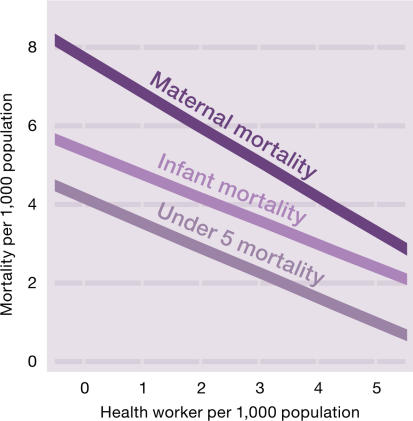
Association between Mortality and Health Worker Density (Illustration: Giovanni Maki, adapted from [9])

The reality, though, is that facilitating the migration of health workers from poor countries contributes to worse health outcomes in these countries. *Human Resources for Health: Overcoming the Crisis* [9], a strategic report of the Joint Learning Initiative (a consortium of over 100 leaders in health), recently analyzed the global workforce [9]. The analysis considered the impact that the global distribution of health workers will have on reaching the health-related Millennium Development Goals. The report suggested that the low health worker density in some countries has already had a major impact on maternal and child mortality (Figure 2) [9]. For example, the report states: “the prospects for achieving 80 percent coverage of measles immunization and skilled attendants at birth are greatly enhanced where worker density exceeds 2.5 workers per 1,000 population. Seventy-five countries with 2.5 billion people are below this minimum threshold.” The report suggests that low health worker density has a particularly marked effect on maternal deaths: a 10% increase in the density of the health workforce is correlated with about a 5% decline in maternal mortality [9]. This strong effect of worker density on maternal health may be due to the fact that highly trained personnel are essential for emergency obstetric services.

Thus, policies and actions that reduce medical and nursing school intake in poor countries while facilitating entry visas into rich countries for physicians and nurses from these same poor countries may be responsible for the deaths of thousands of African children and women. Moral arguments must therefore be used to create policies that moderate the loss of trained health workers from poor countries and stop the medical training subsidies they make to rich countries.

Recruiting countries are much better equipped than source countries to increase their own physician supply and moderate the level of migration. Discussions and debates about health worker migration have, however, also pointed to poor governance and management in source countries as major “push” factors. Strong “pushes” will still exist, even if richer countries control health worker migration in the same way as they have tried to control general immigration.

There are many different policy options for tackling the migration of health professionals', but what works for one country may not work for another, and indeed, what works for nurses may not work for physicians. For example, a Jamaican nursing agency opened an office in Ghana in the 1990s, and Ghana was able to negotiate and agree on the numbers of Ghanaian nurses that Jamaica could recruit—which mitigated the effect of migration. In contrast, doctors tend to seek jobs abroad independently.

Effective international agreements on managing recruitment seem only to work when both source and recipient countries are developing countries. For example, South Africa has been successful at banning recruitment from within Africa, but richer countries opt for voluntary “codes of conduct” that are often quite ineffective [10].

## Conclusion

The migration of physicians and other trained health professionals undermines the ability of developing countries to meet agreed Millennium Development Goals and creates untenable health conditions for the poorer sections of their populations. Developing countries on their own cannot achieve effective moderation of migration and secure the integrity of health services without the cooperation and collaboration of the countries that receive their health workers. An international regimen is needed to manage and moderate the migration of health workers in order to minimize the deleterious effects this has on underdeveloped countries.

Developing countries, on the other hand, need to evolve strategies that reflect their internal needs. This must include designing cadres of health professionals that are trained mainly for the purposes of local needs and are less prone to the attractions of migration.

Countries have different experiences, and each country must develop strategies that reflect the needs of their particular situation. However, the appropriate international environment for managing human resources is necessary if the strategies of developing countries are to achieve meaningful results.
